# Characteristics and needs of physical and mental health among older adult individuals with disabilities under the background of smart healthcare: according to data from the China family panel studies

**DOI:** 10.3389/fpubh.2025.1475466

**Published:** 2025-02-21

**Authors:** Qianlingzi Zou, Xin Guan, Xiaojing Cao, Huangying Gu, Caimeng Wang, Xin Ming, Rongyong Li

**Affiliations:** ^1^Northeast Asian Studies College, Jilin University, Changchun, China; ^2^Guangzhou Xinhua University, Dongguan, China; ^3^Master of Business Administration, London Metropolitan University, London, United Kingdom; ^4^School of Economics, Minzu University of China, Beijing, China; ^5^School of Management, Guangzhou University, Guangzhou, China; ^6^School of Public Administration, Guangzhou University, Guangzhou, China; ^7^China Resources Land Limited, Wuhan, China

**Keywords:** smart healthcare, older adult, disability, physical and mental health, China family panel studies

## Abstract

**Objective:**

This study aimed to utilize data from the China family panel studies (CFPS) to systematically analyze the physical and mental health characteristics and needs of older adult individuals with disabilities under the context of smart healthcare, aiming to provide scientific evidence for relevant policy formulation.

**Methods:**

Data were derived from the CFPS surveys conducted by Peking University’s China Social Sciences between 2021 and 2022. Older adult individuals aged 65 and above requiring assistance in activities of daily living (ADL) or instrumental activities of daily living (IADL) were included. Descriptive statistics, univariate analysis, and multivariate analysis were employed to analyze the basic characteristics, physical and mental health status, influencing factors, and corresponding needs of older adult individuals with disabilities.

**Results:**

Samples aged between 65 and 80, widowed or divorced, demonstrated higher physical health scores (*p* < 0.05); samples with education at college level or above, residing in urban areas, free from chronic diseases, with annual income exceeding 100,000 RMB, receiving daily family support, frequently accessing community services, and regularly using smart medical devices exhibited greatly higher levels of physical health, mental health, and overall scores on the short form health survey (SF-36) (*p* < 0.05). Factors such as younger age, absence of chronic diseases, higher economic status, daily family support, frequent access to community services, and regular use of smart medical devices exerted favorable effects on the physical health status of older adult individuals with disabilities (*p* < 0.05); absence of chronic diseases, urban residence, higher economic status, daily family support, and frequent access to community services were found to positively influence the mental health status of older adult individuals with disabilities (*p* < 0.05).

**Conclusion:**

The physical and mental health status of older adult individuals with disabilities is influenced by various factors, including social support, economic conditions, and utilization of medical resources. These factors constitute significant determinants in improving the physical and mental health of older adult individuals with disabilities and represent key needs for their betterment.

## 1 Introduction

With the accelerated global aging process, particularly evident in China, the older adult population has been rapidly expanding. According to statistics, the population aged 65 and above in China surpassed 200 million in 2023, accounting for over 14% of the total population (1). Among them, the number of older adult individuals with disabilities (those requiring assistance in daily activities) continues to rise. One of the most immediate challenges brought about by an aging society is effectively managing and maintaining the health of the older adult, especially concerning the physical and mental well-being of those with disabilities (2).

In recent years, with the rapid development of information technology and medical technology, smart healthcare has emerged as a crucial innovation domain. Smart healthcare refers to the utilization of advanced technological means such as big data analytics, artificial intelligence, the Internet of Things (IoT), wearable devices, and telemedicine to provide precise, efficient, and personalized medical services to individuals (3). These technologies not only contribute to enhancing the quality and efficiency of medical services but also, through continuous health monitoring and management, improve patients’ health status and quality of life (4).

Older adult individuals with disabilities typically face various health issues, including chronic diseases, cognitive decline, and psychological health problems (5–7). Due to the decline in physiological functions, they are more susceptible to multiple chronic conditions, while psychological health issues such as depression and anxiety are also prevalent in this demographic (8). These challenges not only diminish the quality of life (QoL) for the older adult but also impose burdens on their families and society at large. However, the adoption of smart healthcare technology in managing the health of older adult individuals with disabilities offers new avenues to address these issues. For instance, real-time monitoring of older adult individuals’ health status through wearable devices and IoT technology allows for the timely detection and management of health issues. Moreover, personalized health management plans can be developed for the older adult using big data and artificial intelligence technologies, thereby enhancing the precision and effectiveness of medical services (9). Furthermore, the adoption of telemedicine technology enables older adult individuals to access high-quality medical services from the comfort of their homes, reducing the difficulties and inconveniences associated with seeking medical care (10).

Despite the tremendous potential of smart healthcare in managing the health of older adult individuals with disabilities, research in this field remains limited. Particularly in China, there is still insufficient research on the physical and mental health characteristics of older adult individuals with disabilities and their specific needs within the context of smart healthcare. Therefore, this study aimed to delve into the physical and mental health characteristics of older adult individuals with disabilities and their specific needs in a smart healthcare environment based on data from the China Family Panel Studies. The objective was to provide insights for the optimization of smart healthcare systems and the formulation of relevant policies.

## 2 Literature review

With the exacerbation of China’s aging population, the physical and mental health issues of older adult individuals with disabilities are becoming increasingly prominent. Older adult individuals often suffer from various chronic diseases, as Maresova et al. (11) suggested, the increase in chronic and degenerative diseases is associated with the continuous growth of the older adult population. Thakur et al. (12), through statistical analysis, found that patients aged 60 and above have a higher prevalence of chronic diseases, mainly including hypertension (60.64%), diabetes (35.8%), cancer (28.38%), and coronary artery disease (22.58%), with over half (52.9%) of the patients having two or more diseases. With advancing age, physiological decline in organ function may lead to issues such as memory decline, decreased attention span, and slowed thinking speed, potentially progressing to cognitive impairment or dementia (13). The emergence of psychological health issues in the older adult is closely related to physiological, social, and psychological factors (14). As individuals age, psychological disorders related to physiological changes may occur, such as late-life depression and late-life anxiety disorders. Furthermore, factors such as reduced social support, loss of relatives or friends, and declining physical function may exacerbate psychological health issues in the older adult. These psychological health issues not only affect the quality of life for older adult individuals but may also increase their risk of developing other chronic diseases.

Smart healthcare involves leveraging advanced information technology and intelligent algorithms to enhance healthcare services. Its adoptions in the medical field mainly include remote healthcare services, health monitoring and tracking, personalized medicine, medical imaging diagnosis, medical robotics, smart drug management, medical big data analysis, among others. It provides older adult individuals with continuous health monitoring and personalized health management services. For instance, Tiersen et al. (15) proposed remote monitoring and intervention for older adult dementia patients. Ji and Kim (16) suggested that smart healthcare not only improves the efficiency and quality of medical services but also enhances the self-management capabilities and quality of life for the older adult. Older adult individuals with disabilities often have specific needs in medical services, daily care, and psychological support due to physiological and psychological deficiencies (17). Research indicated that these older adult individuals require comprehensive care services, including medical, rehabilitative, nursing, and psychological counseling. Smart healthcare, through personalized health management plans, can meet the diverse needs of the older adult (18). For example, smart health monitoring devices can remotely monitor the health status of older adult individuals, detect abnormalities in a timely manner, and intervene accordingly; smart rehabilitation devices can assist older adult individuals in rehabilitation training, thereby improving their quality of life and self-care abilities; smart mental health adoptions can provide personalized psychological support and counseling services, helping older adult individuals manage emotions and psychological health issues. Overall, the development of smart healthcare presents new opportunities and possibilities for meeting the diverse needs of older adult individuals with disabilities, potentially enhancing their health status and quality of life.

**Figure 1:**
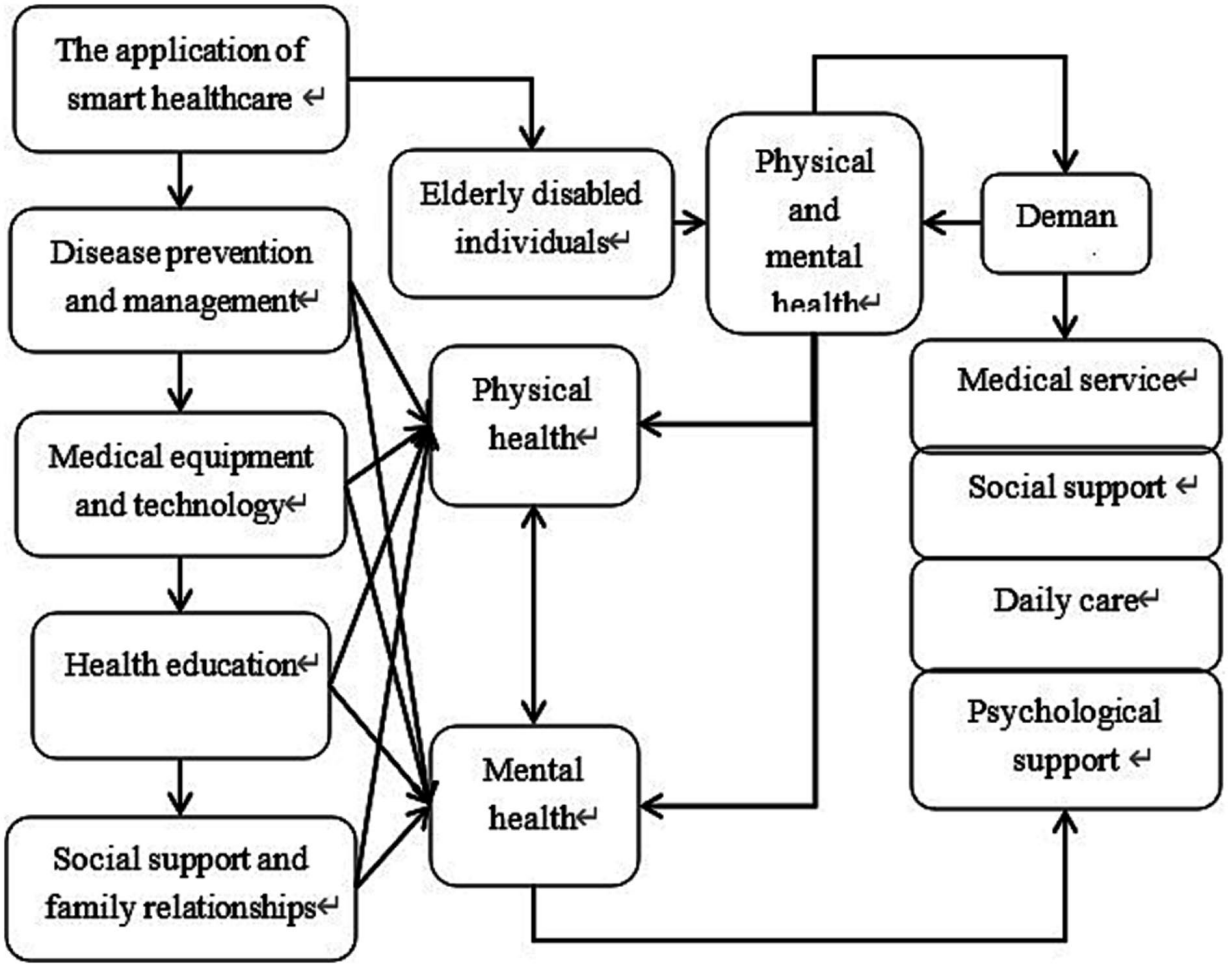
Path diagram of the relationship between smart healthcare and the physical and mental health as well as the needs of older adult individuals with disabilities.

The aforementioned pathways of smart healthcare, including disease prevention and management, medical technologies and devices, and health education, influence the physical and mental health of older adult individuals with disabilities. Physical and mental health can be further divided into two aspects: physical health and psychological health, both of which mutually influence each other. The status of physical and mental health directly impacts the needs of older adult individuals, encompassing requirements for healthcare and psychological support. Additionally, social support and family relationships play significant roles in shaping physical and psychological health, thereby affecting the needs of older adult individuals. In light of this, a path analysis diagram was constructed (Figure 1). However, there is currently insufficient research on the impact of smart healthcare adoptions on the physical and mental health characteristics of older adult individuals in China.

## 3 Research methodology

This study, based on data from the China family panel studies (CFPS), employed a quantitative research approach to investigate the physical and mental health characteristics and needs of older adult individuals with disabilities through statistical analysis.

### 3.1 Data sources

The data for this study were obtained from the CFPS conducted between 2021 and 2023. CFPS, administered by the China Social Science Survey Center at Peking University, encompasses a nationally representative sample from mainland China. The study tracked and collected information on the use of smart healthcare devices, household economy, educational status, health conditions, social support, and utilization of medical services. CFPS data were characterized by extensive representativeness and high quality, providing a reliable data foundation for investigating the health status and needs of older adult individuals in China.

### 3.2 Sample selection

The study focused on older adult individuals with disabilities. Accordingly, the specific selection criteria for the sample were outlined as follows:

(1) Participants must be aged 65 years or older.
(2) Participants must meet the disability criteria, which were assessed based on activities of daily living (ADL) and instrumental activities of daily living (IADL). Specific indicators included dressing, bathing, eating, getting in and out of bed, toileting, indoor mobility, using the telephone, shopping, meal preparation, housekeeping, medication management, and financial management. Each activity was scored based on the respondent’s level of independence, typically categorized as completely independent (no assistance needed), requiring partial assistance, or completely dependent on others. Specifically, if a respondent required partial assistance or was completely dependent on others in one or more ADL or IADL activities, they were classified as older adult individuals with disabilities.
(3) Sample data must be complete, particularly with regard to key variables such as gender, age, ADL assessment, health assessment, psychological assessment, and chronic disease information with no missing values.

Based on the above criteria, older adult individuals with disabilities who met the defined criteria were selected. Samples with missing or incomplete data were excluded, resulting in a final effective sample size of 7,839 cases.

### 3.3 Research variables

Basic variables included the usage of smart healthcare (mainly types of smart healthcare), gender, age, marital status, education level, living situation, economic status, presence of chronic diseases, family support, and community service utilization.

Physical and mental health characteristics were as follows. In this study, all samples underwent assessment of physical and mental health using the short form health survey (SF-36), which has demonstrated good reliability and validity (19). The SF-36 questionnaire was used to evaluate an individual’s health status and quality of life. It comprises eight health concepts, namely physical functioning (PF), role physical (RP), bodily pain (BP), general health (GH), vitality (VT), social functioning (SF), role emotional (RE), and mental health (MH). Responses to each item were rated on a Likert scale, and then the scores for each item were converted to standardized scores ranging from 0 to 100. Higher scores indicate better quality of life for older adult individuals, while lower scores indicate poorer quality of life. Standardized scores are categorized into three levels: poor (0–60 points), fair to moderate (61–95 points), and excellent (96–100 points).

### 3.4 Statistical methods

The data were processed and analyzed using SPSS 20.0. Descriptive statistical analysis was employed to characterize the samples, with categorical data presented as percentages (%) and analyzed using the chi-square test. Continuous data were denoted as means ± standard deviations, and independent t-tests were conducted for comparison. Additionally, multiple-factor logistic regression analysis was employed to analyze the factors influencing the physical and mental health of older adult individuals with disabilities. Statistical significance was set at *p* < 0.05 to indicate the presence of differences.

## 4 Results

### 4.1 Basic situation statistics

The study statistically analyzed the demographic characteristics of the collected samples, including the usage of smart healthcare (primarily types and frequency), gender, age, marital status, education level, residence, economic status, presence of chronic diseases, family support, and community service utilization, as detailed in Table 1. It can be observed that older adult individuals with disabilities who use smart healthcare were predominantly married (spouse alive) (52.97%), residing in urban areas (63.96%), having an education level of junior high school or above (76.98%), and having social security coverage (85.04%).

**Table 1:** Basic statistics of research samples.

Factor		Number of cases (*n*)	Proportion (%)
Sex	Male	3,402	43.40
	Female	4,437	56.60
Age (years old)	65 ~ 70	1,959	24.99
	70 ~ 75	2,196	28.01
	75 ~ 80	1,881	24.00
	80~	1,803	23.00
Marriage	Unmarried	392	5.00
	Married (Spouse Alive)	4,152	52.97
	Widowed	2,115	26.98
	Divorced	1,180	15.05
Educational level	Illiterate	783	9.99
	Primary school or below	1,021	13.02
	Junior high school	2,115	26.98
	Senior high school / Vocational school	1,568	20.00
	College or above	2,352	30.00
Residence	Urban	5,014	63.96
	Rural	2,825	36.04
Chronic diseases	Yes	4,703	59.99
	No	3,136	40.01
Economy (annual income)	<1 W	1,176	15.00
	1 ~ 5 W	1,959	24.99
	5 ~ 10 W	1,568	20.00
	>10 W	3,136	40.01
Family support	Every day	3,920	50.01
	Often	2,352	30.00
	Occasionally	1,098	14.01
	Very rarely	469	5.98
Community service	Frequently	3,136	40.01
	Occasionally	2,352	30.00
	Almost never	2,351	29.99
Smart healthcare usage	Remote medical care	3,136	40.01
	Health monitoring devices	2,352	30.00
	Online medical consultation	3,136	40.01
	Electronic health record management	1,568	20.00
	Smart medication management	1,568	20.00
	Virtual health assistant	1,176	15.00
	Health information platform	2,352	30.00
	Online pharmacy services	1,176	15.00
Usage frequency	Often	3,136	40.01
	Occasionally	3,135	39.99
	Very rarely	1,568	20.00

### 4.2 SF-36 scores under different genders

**Figure 2:**
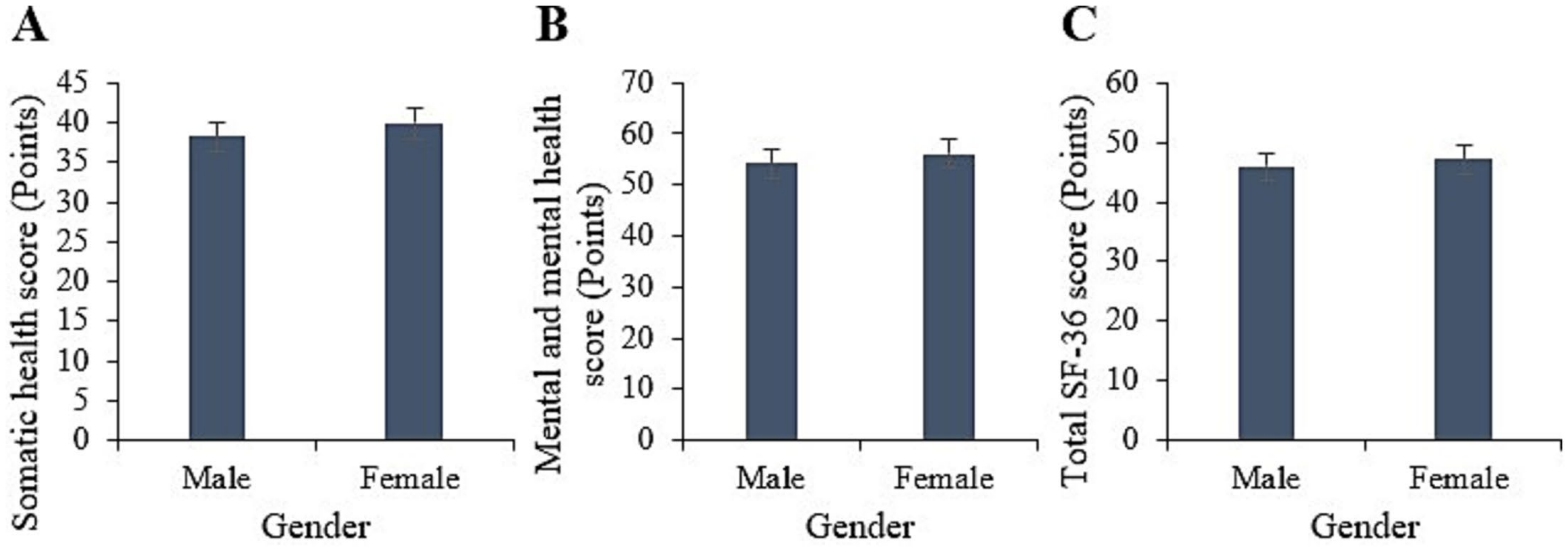
Comparison of physical health **(A)**, mental health **(B)**, and total SF-36 scores **(C)** between males and females.

Statistically, the physical health score for male participants in this study was (38.22 ± 8.65), with a mental health score of (54.23 ± 8.09), and a total SF-36 score of (45.98 ± 7.33); whereas for female participants, the physical health score was (39.88 ± 7.35), with a mental health score of (56.11 ± 8.22), and a total SF-36 score of (47.13 ± 7.01). Upon comparison, neglectable differences were observed between male and female participants in terms of physical health, mental health, and total SF-36 scores (*p* > 0.05) (Figure 2).

### 4.3 SF-36 scores at different ages

**Figure 3:**
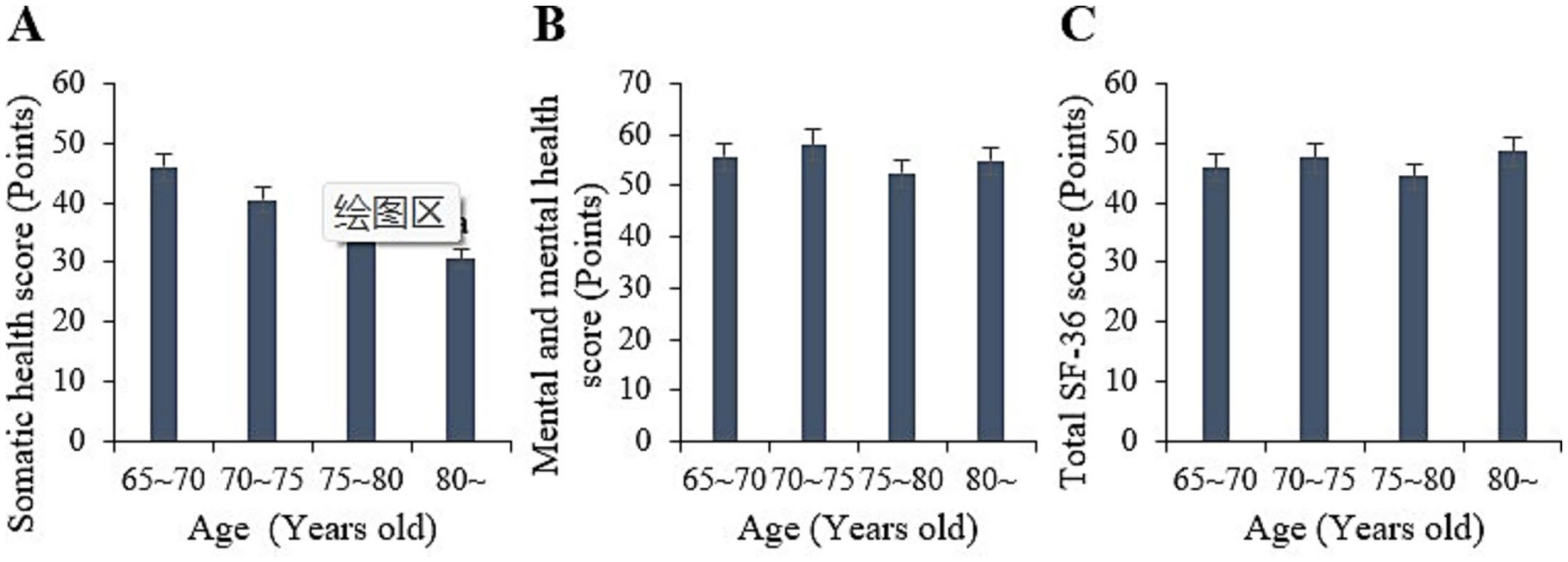
Comparison of physical health **(A)**, mental health **(B)**, and total SF-36 scores **(C)** among samples in different age groups. (^a^*p* < 0.05 vs. samples aged 65–80).

Statistically, among the samples aged 65 to 70 years in this study, the physical, mental, and overall SF-36 scores were (45.96 ± 6.17), (55.57 ± 7.93), and (45.82 ± 7.11) points, respectively. For the age group of 70 to 75 years, the corresponding scores were (40.52 ± 7.03), (57.92 ± 7.37), and (47.5 ± 7.00) points. For the 75 to 80 years age group, the scores were (39.14 ± 5.98), (52.38 ± 9.28), and (44.34 ± 7.7) points, respectively. For those aged 80 years and above, the scores were (30.58 ± 6.83), (54.81 ± 9.82), and (48.56 ± 6.09) points, respectively. Upon comparison, the physical health scores of the 65 to 80 years age group were greatly higher versus those aged 80 years and above (*p* < 0.05). However, neglectable differences were observed in the mental health and overall SF-36 scores among the various age groups (*p* > 0.05) (Figure 3).

### 4.4 SF-36 scores under different marriages

**Figure 4:**
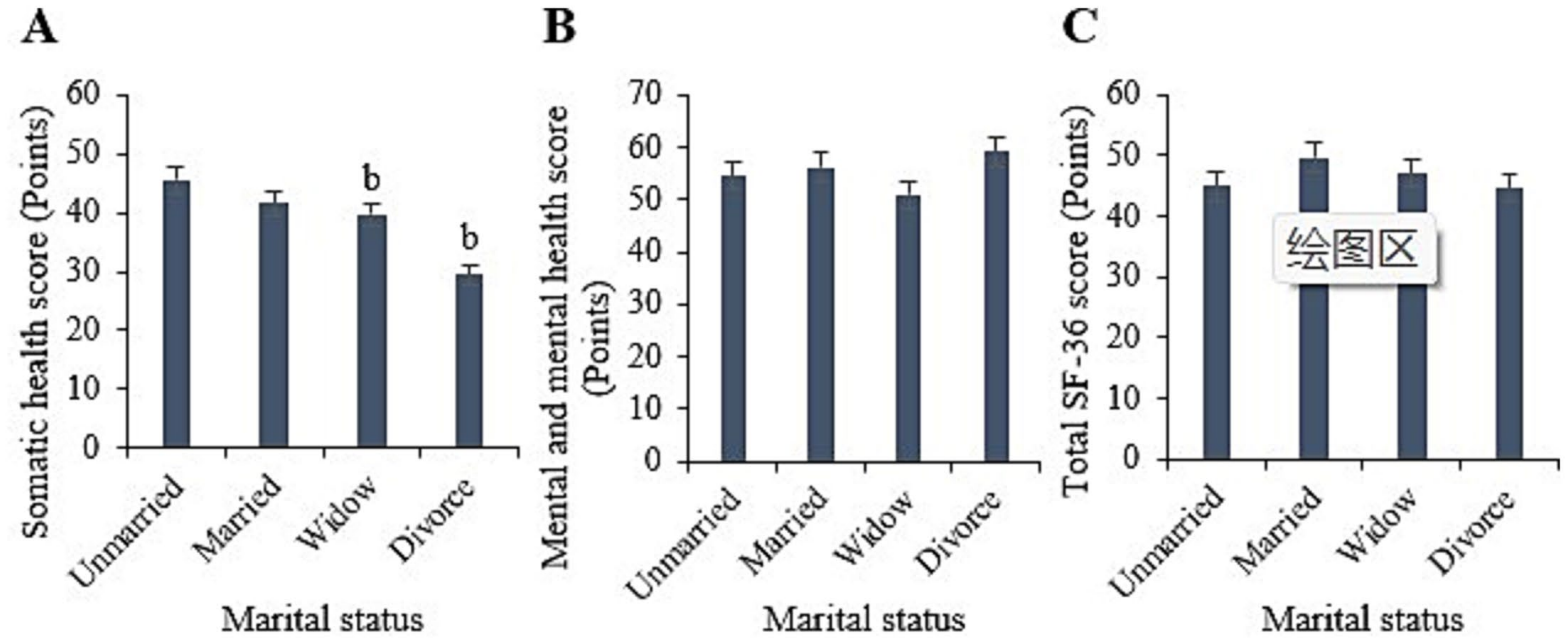
Comparison of physical health **(A)**, mental health **(B)**, and total SF-36 scores **(C)** among samples with different marital statuses. (^b^*p* < 0.05 vs. samples of unmarried status).

Statistically, in this study, the physical, mental, and overall SF-36 scores for the unmarried samples were (45.6 ± 7.00), (54.55 ± 7.99), and (44.92 ± 6.86) points, respectively. For the married samples, the scores were (41.59 ± 6.22), (56.12 ± 9.04), and (49.68 ± 7.88) points, respectively. For the widowed samples, the scores were (39.62 ± 5.67), (50.85 ± 9.03), and (47.06 ± 7.30) points, respectively. For the divorced samples, the scores were (29.39 ± 5.09), (59.16 ± 9.31), and (44.56 ± 6.78) points, respectively. Upon comparison, it was observed that the physical health scores of the widowed and divorced samples were notably inferior to those of the unmarried samples (*p* < 0.05). However, the mental health and overall SF-36 scores differed slightly across different marital statuses (*p* > 0.05) (Figure 4).

### 4.5 SF-36 ratings under different cultural levels

**Figure 5:**
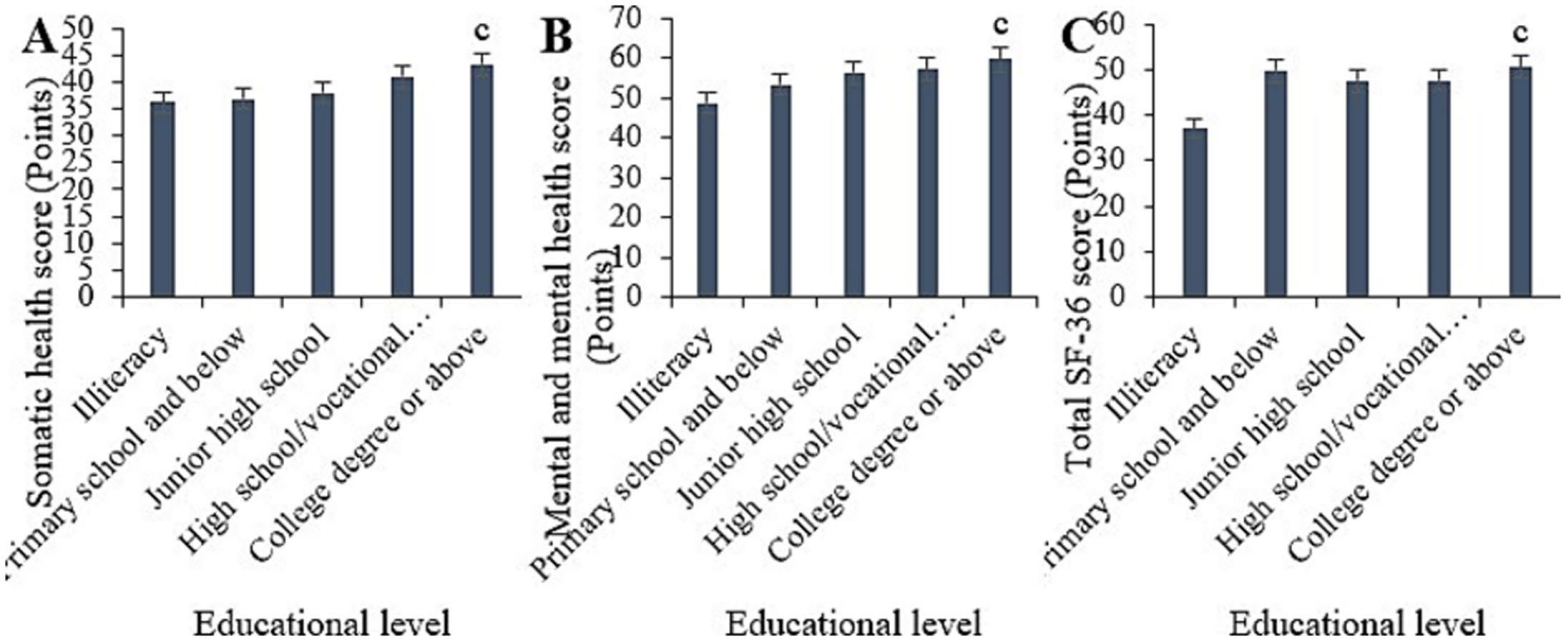
Comparison of physical health **(A)**, mental health **(B)**, and total SF-36 scores **(C)** among samples with different educational levels. (^c^*p* < 0.05 vs. samples of illiterate and primary education or below).

Statistically, in this study, the physical, mental, and overall SF-36 scores for illiterate samples were (36.22 ± 5.27), (48.81 ± 7.03), and (37.13 ± 6.36) points, respectively. For samples with primary education or below, the scores were (36.89 ± 6.04), (53.43 ± 9.04), and (49.84 ± 7.66) points, respectively. For samples with junior high school education, the scores were (37.94 ± 5.33), (56.43 ± 9.37), and (47.58 ± 7.08) points, respectively. For samples with high school/technical secondary school education, the scores were (40.97 ± 5.45), (57.35 ± 9.18), and (47.65 ± 6.67) points, respectively. For samples with college education or above, the scores were (43.23 ± 5.32), (59.83 ± 9.00), and (50.58 ± 8.64) points, respectively. Upon comparison, it was observed that the physical, mental, and overall SF-36 scores of samples with college education or above were markedly higher than those of illiterate samples and samples with primary education or below (*p* < 0.05) (Figure 5).

### 4.6 SF-36 ratings under different residential conditions

**Figure 6:**
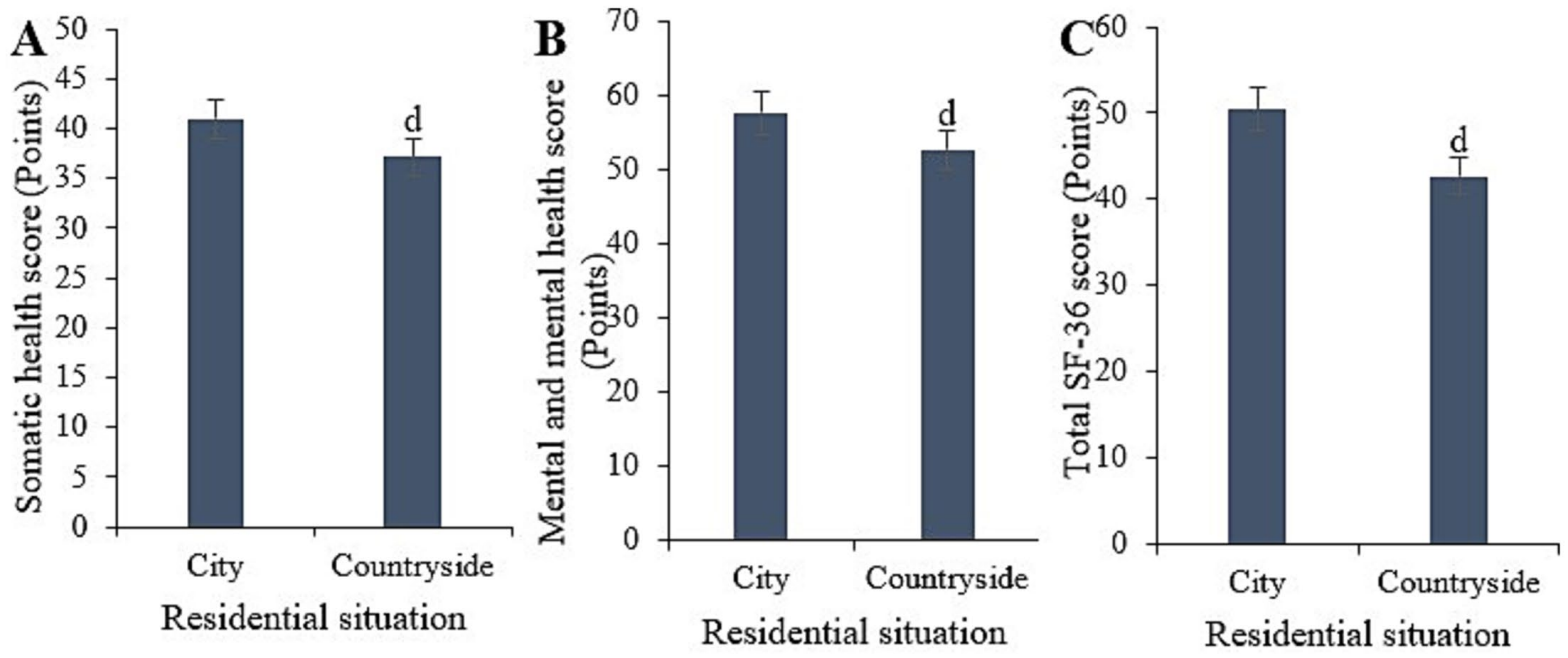
Comparison of physical health **(A)**, mental health **(B)**, and total SF-36 scores **(C)** among samples with different cultural residence statuses. (^d^*p* < 0.05 vs. samples from urban areas).

Statistically, in this study, the physical health score for urban samples was (40.95 ± 6.26), with a mental health score of (57.68 ± 7.16), and a total SF-36 score of (50.41 ± 8.03). In contrast, for rural samples, the physical health score was (37.15 ± 4.09), with a mental health score of (52.66 ± 7.21), and a total SF-36 score of (42.7 ± 6.38). Upon comparison, it was evident that individuals residing in urban areas had substantially higher physical health, mental health, and overall SF-36 scores relative to those living in rural areas (*p* < 0.05) (Figure 6).

### 4.7 SF-36 score under different chronic disease conditions

**Figure 7:**
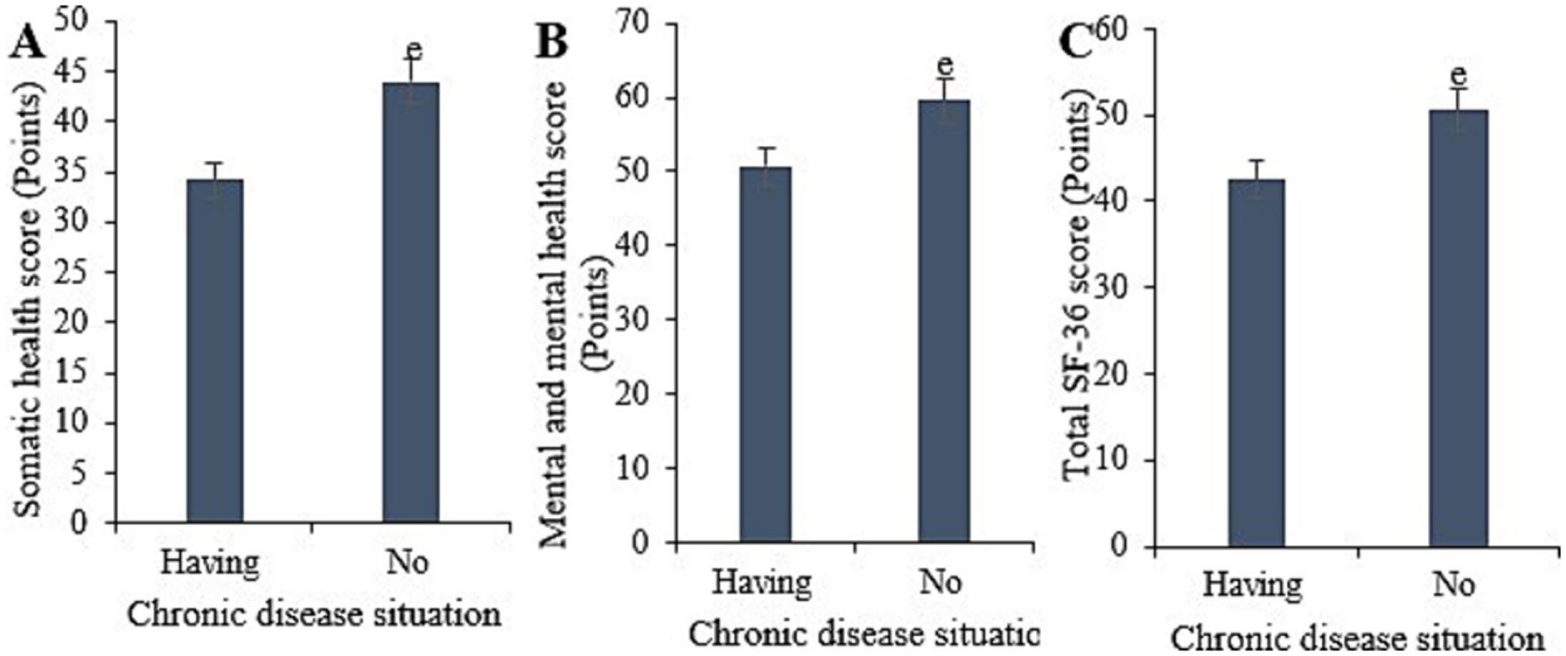
Comparison of physical health **(A)**, mental health **(B)**, and total SF-36 scores **(C)** among samples with different chronic disease statuses. (^e^*p* < 0.05 vs. samples with chronic diseases).

Statistically, in this study, the physical health score for samples with chronic diseases was (34.18 ± 6.00), with a mental health score of (50.69 ± 7.11), and a total SF-36 score of (42.52 ± 7.27). Conversely, for samples without chronic diseases, the physical health score was (43.92 ± 7.34), with a mental health score of (59.65 ± 9.32), and a total SF-36 score of (50.59 ± 7.48). Upon comparison, it was evident that samples without chronic diseases exhibited notably higher physical health, mental health, and overall SF-36 scores versus those with chronic diseases (*p* < 0.05) (Figure 7).

### 4.8 SF-36 ratings under different economic conditions

**Figure 8:**
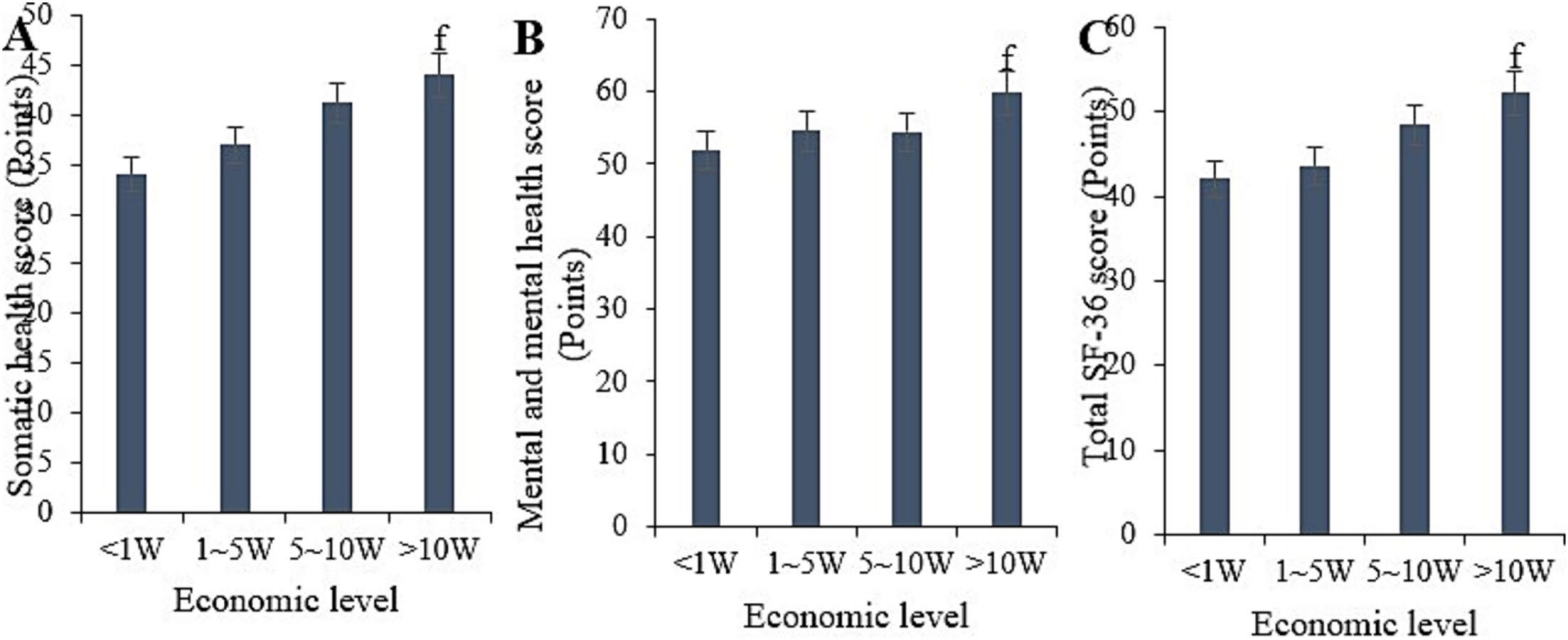
Comparison of physical health **(A)**, mental health **(B)**, and total SF-36 scores **(C)** among samples with different economic statuses. (^f^*p* < 0.05 vs. samples with an income of less than 1 W).

Based on statistical analysis, the physical health score for the sample with an annual income below 10,000 yuan was (34 ± 5.28), the mental health score was (51.86 ± 7.09), and the overall SF-36 score was (42.05 ± 6.38). For the sample with an annual income between 10,000 and 50,000 yuan, the scores were (37 ± 5.16), (54.59 ± 9.48), and (43.56 ± 6.47), respectively. For the sample with an annual income between 50,000 and 100,000 yuan, the scores were (41.2 ± 6.84), (54.43 ± 8.98), and (48.4 ± 6.59), respectively. For the sample with an annual income above 100,000 yuan, the scores were (44 ± 5.84), (59.8 ± 9.98), and (52.21 ± 7.39), respectively. Comparative analysis indicated that the physical health, mental health, and overall SF-36 scores for the sample with an annual income above 100,000 yuan were considerably higher than those for the sample with an annual income below 10,000 yuan (*p* < 0.05) (Figure 8).

### 4.9 SF-36 scores under different family support conditions

**Figure 9:**
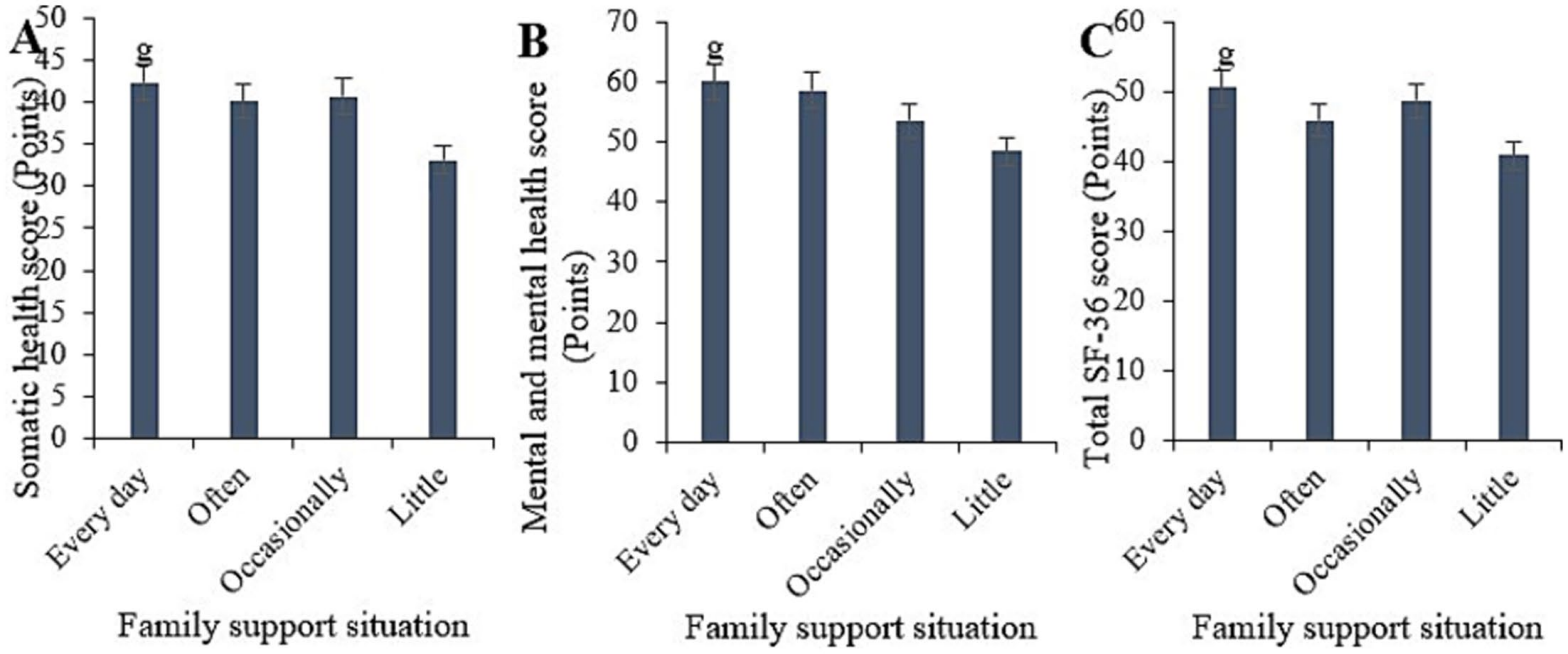
Comparison of physical health **(A)**, mental health **(B)**, and total SF-36 scores **(C)** among samples with different levels of family support. (^g^*p* < 0.05 vs. samples with low family support).

Based on statistical analysis, the physical health score for the sample receiving daily family support was (42.31 ± 5.12), the mental health score was (60.1 ± 7.11), and the overall SF-36 score was (50.6 ± 6.02). For the sample receiving frequent support, the scores were (40.07 ± 5.23), (58.61 ± 9.40), and (45.93 ± 6.13), respectively. For the sample receiving occasional support, the scores were (40.71 ± 6.04), (53.54 ± 8.18), and (48.75 ± 6.33), respectively. For the sample receiving little support, the scores were (33.11 ± 4.12), (48.43 ± 6.12), and (40.94 ± 5.66), respectively. Comparative analysis indicated that the physical health, mental health, and overall SF-36 scores for the sample receiving daily family support were notably superior to those for the sample receiving little support (*p* < 0.05) (Figure 9).

### 4.10 SF-36 ratings under different community service situations

**Figure 10:**
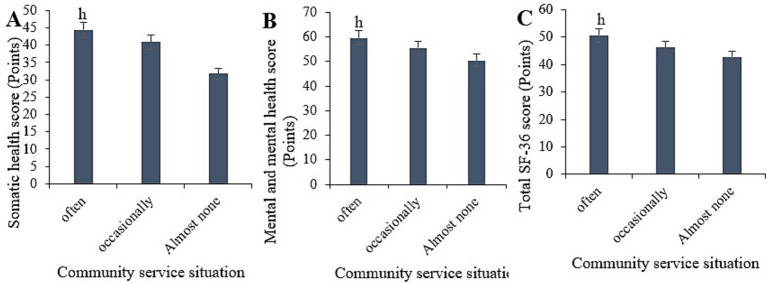
Comparison of physical health **(A)**, mental health **(B)**, and total SF-36 scores **(C)** among samples with different levels of family support. (^h^*p* < 0.05 vs. samples with minimal community support).

Based on statistical analysis, the physical health score for the sample frequently receiving community services was (44.48 ± 5.09), the mental health score was (59.55 ± 7.03), and the overall SF-36 score was (50.62 ± 6.28). For the sample occasionally receiving community services, the scores were (40.88 ± 5.88), (55.49 ± 7.89), and (46.27 ± 6.74), respectively. For the sample rarely receiving community services, the scores were (31.79 ± 4.78), (50.47 ± 7.56), and (42.78 ± 6.22), respectively. Comparative analysis indicated that the physical health, mental health, and overall SF-36 scores for the sample frequently receiving community services were substantially superior to those for the sample rarely receiving community services (*p* < 0.05) (Figure 10).

### 4.11 SF-36 scores under different types of smart healthcare

**Figure 11:**
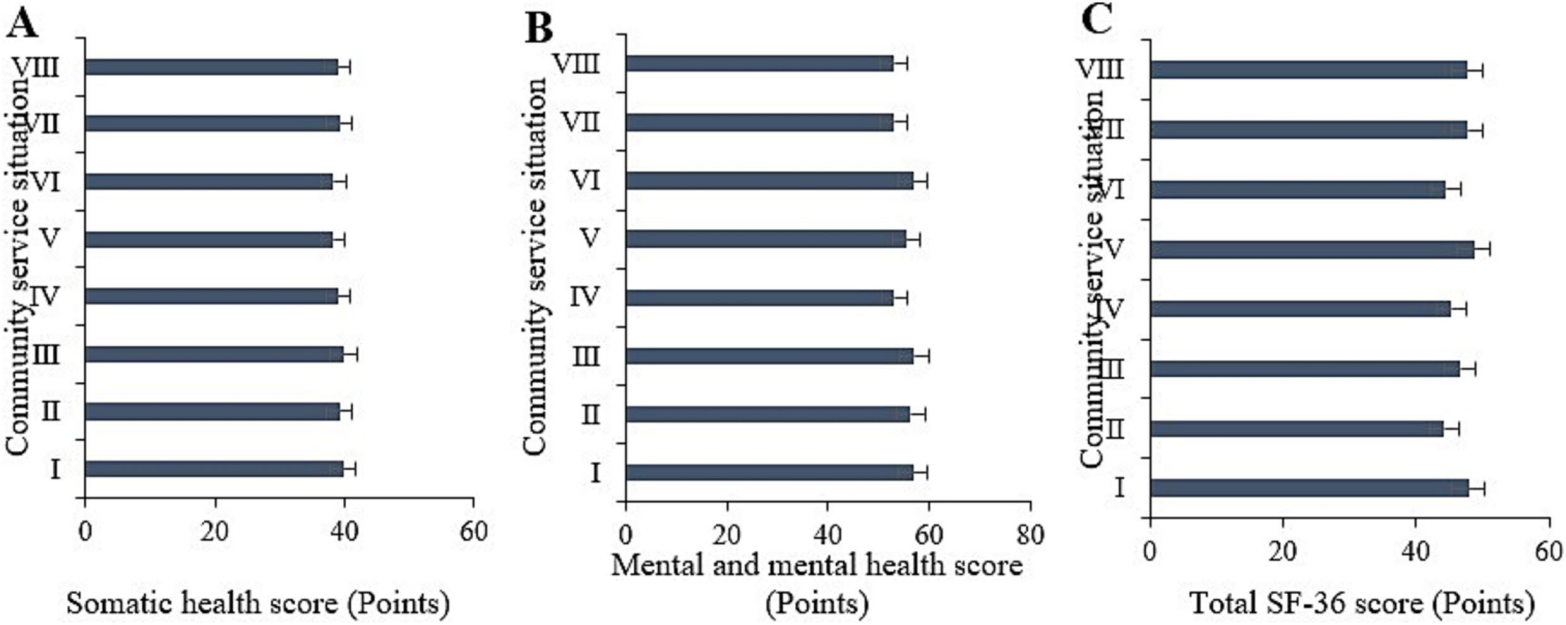
Comparison of physical health **(A)**, mental health **(B)**, and total SF-36 scores **(C)** among samples with different types of smart healthcare usage. (I ~ VIII represent remote healthcare, health monitoring devices, online medical consultation, electronic health record management, smart medication management, virtual health assistants, health information platforms, online pharmacy services, respectively).

The study statistically analyzed the physical health, mental health, and overall SF-36 scores under different types of smart healthcare (Figure 11). Comparative analysis indicated neglectable differences in the physical health, mental health, and overall SF-36 scores among those using telemedicine, health monitoring devices, online medical consultations, electronic health record management, smart medication management, virtual health assistants, health information platforms, and online medication purchasing services (*p* > 0.05).

### 4.12 Scoring of SF-36 under different usage frequencies of smart healthcare

**Figure 12:**
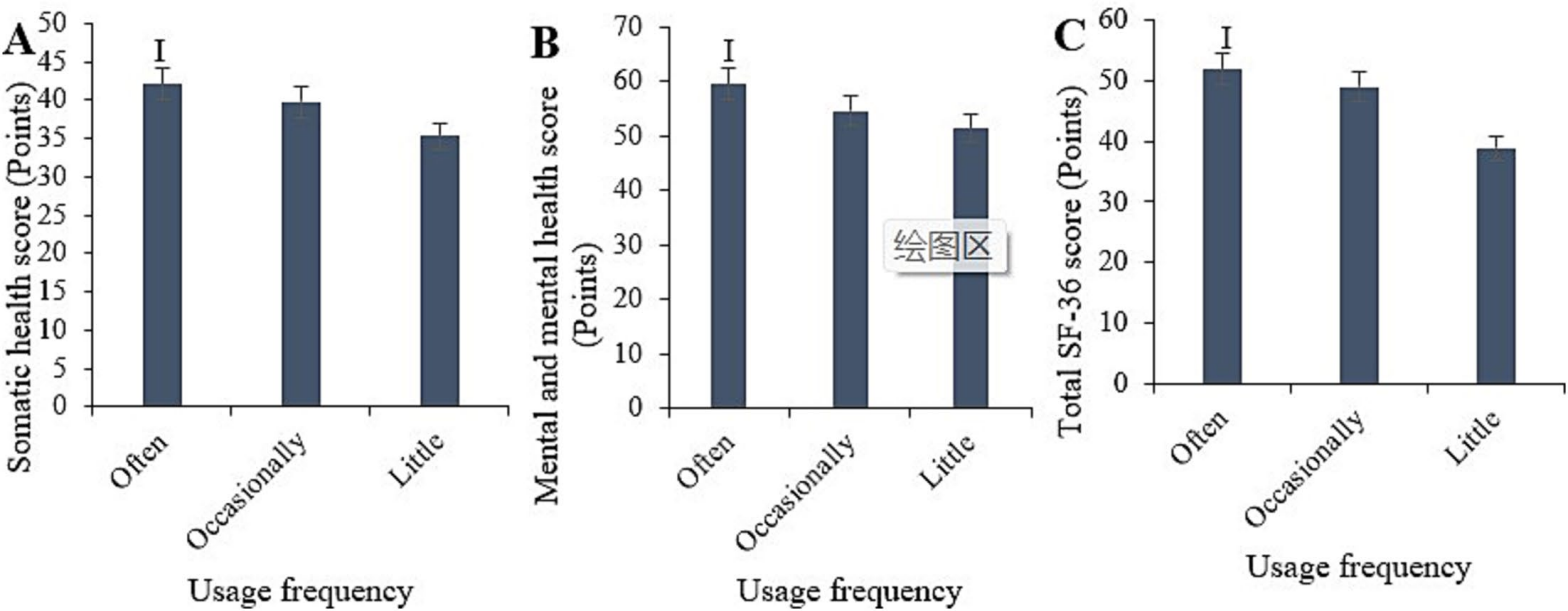
Comparison of physical health **(A)**, mental health **(B)**, and total SF-36 scores **(C)** among samples with different frequencies of smart healthcare usage. (^i^*p* < 0.05 vs. samples with infrequent usage).

The study statistically analyzed the physical health, mental health, and overall SF-36 scores under different frequencies of smart healthcare usage (Figure 12). The scores for frequent users were (44.48 ± 5.09) for physical health, (59.55 ± 7.03) for mental health, and (50.62 ± 6.28) for overall SF-36. For occasional users, the scores were (40.88 ± 5.88), (55.49 ± 7.89), and (46.27 ± 6.74), respectively. For rare users, the scores were (31.79 ± 4.78), (50.47 ± 7.56), and (42.78 ± 6.22), respectively. Comparative analysis indicated that the physical health, mental health, and overall SF-36 scores for frequent users were markedly superior to those for rare users (*p* < 0.05).

### 4.13 Multivariate logistic analysis on the physical health of older adult disabled individuals

The study used the variables identified with significant differences from the aforementioned univariate analysis to examine factors influencing the physical health of older adult individuals with disabilities. The results indicated that younger age, absence of chronic diseases, higher economic status, daily family support, frequent community services, and regular use of smart healthcare devices positively impacted the physical health of older adult individuals with disabilities (*p* < 0.05) (Table 2).

**Table 2:** Multivariate logistic analysis on the impact of physical health in older adult disabled individuals.

Factor	*p*	*t*	*OR*	*95%CI*
65 ~ 70 years old	0.012	−6.493	0.758	0.372 ~ 0.982
No chronic diseases	0.008	−5.823	0.873	0.621 ~ 0.999
Annual income above 100,000 yuan	0.002	6.825	0.672	0.561 ~ 0.872
Daily family support	0.028	5.933	0.983	0.609 ~ 1.103
Frequent community services	0.011	8.056	0.673	0.493 ~ 0.892
Regular use of smart healthcare devices	0.003	7.944	0.983	0.683 ~ 1.201

### 4.14 Multivariate logistic analysis on the psychological health of older adult disabled individuals

The variables identified with significant differences from the aforementioned univariate analysis were subjected to variable analysis to examine factors influencing the mental health of older adult individuals with disabilities. The results indicated that the absence of chronic diseases, residing in urban areas, higher economic status, daily family support, and frequent community services positively impacted the mental health status of older adult individuals with disabilities (*p* < 0.05) (Table 3).

**Table 3:** Multivariate logistic analysis on the psychological health of older adult disabled individuals.

Factor	*p*	*t*	*OR*	95%*CI*
No chronic diseases	0.015	−5.988	0.773	0.601 ~ 0.983
Living in urban areas	0.039	6.345	0.456	0.309 ~ 0.784
Annual income above 100,000 yuan	0.012	6.821	0.693	0.520 ~ 0.812
Daily family support	0.018	5.909	0.900	0.622 ~ 1.111
Frequent community services	0.010	8.093	0.622	0.423 ~ 0.834

## 5 Discussion

As China’s society continues to age, the health issues of the older adult population with disabilities have increasingly become a focus of attention. Smart healthcare, as a product of the combination of modern medical technology and information technology, has shown tremendous potential in improving health management and medical services for the older adult. This study aimed to explore the physical and mental health characteristics and needs of older adult individuals with disabilities under the background of smart healthcare, with the objective of providing scientific evidence for the formulation of relevant policies and the optimization of services.

The study revealed that individuals aged 80 and above tend to have lower scores on physical health assessments, indicating a declining trend in physical health with advancing age among the older adult. Due to the natural aging process, various bodily functions gradually deteriorate in individuals aged 80 and above, leading to decreased physical strength and immunity, rendering them susceptible to various health issues. Moreover, individuals aged 80 and above are more prone to accumulating multiple chronic diseases, which mutually exacerbate their health burden (20). The physical health scores of widowed and divorced participants were notably inferior to those of unmarried participants (*p* < 0.05), suggesting a great influence of marital status on the health of the older adult, particularly among widowed and divorced individuals. This may be attributed to the psychological stress and loneliness resulting from the loss of a spouse, which adversely affects their health. Conversely, unmarried older adult individuals may have already adapted to living alone, possessing better self-care capabilities and thus relatively better health conditions (21). The physical health, mental health, and overall SF-36 scores of individuals with a college education or higher were markedly superior to those of individuals with no formal education, or with primary education or below (*p* < 0.05). This is attributed to the fact that older adult individuals with higher levels of education often possess better health awareness and knowledge, enabling them to understand and adhere to health advice and treatment regimens, thereby maintaining better health status. Furthermore, older adult individuals with higher levels of education typically have better socioeconomic status, gaining access to more health resources and support, and often exhibiting a higher acceptance of smart healthcare adoptions (22). Additionally, samples with higher economic status, more frequent use of smart healthcare, and urban residency demonstrated better physical and mental health. This is because higher economic status and urban residence are associated with relatively richer smart healthcare resources, health services, and living facilities, making it easier for the older adult to access high-quality medical care and community services, thus favoring the adoption of smart healthcare devices. However, rural areas exhibit certain deficiencies in these aspects, which are not conducive to the management and monitoring of the health of the older adult, thereby affecting their physical and mental well-being (23). In the study, the physical and mental health scores of the sample without chronic diseases were significantly higher than those of the sample with chronic diseases. Chronic diseases such as diabetes, hypertension, and heart disease require long-term management and treatment, which not only increase physical burden but also may lead to a series of complications (24). Moreover, the uncertainty during the long-term illness and treatment process can easily lead to psychological issues such as anxiety and depression (25). Patients with chronic diseases need to constantly cope with changes in their condition and adjustments to treatment plans, resulting in a heavier psychological burden. Older adult individuals with advanced age and disability often experience compromised physical health due to factors such as chronic illnesses and reduced bodily functions. The loss of independence, decline in quality of life, and social isolation resulting from disability contribute to the development of depressive and anxious symptoms among many older adult individuals with advanced age and disability (26). The care and companionship of family members can alleviate the feelings of loneliness and psychological pressure among older adult individuals, providing emotional comfort. Material support ensures that the older adult can access sufficient nutrition, medical care, and daily assistance, thereby helping to maintain and improve their physical health. Community health services, including regular health check-ups, disease prevention, and health education, contribute to the timely detection and management of health issues, thereby enhancing the health status of older adult individuals. Social activities can reduce the feelings of loneliness and social isolation among the older adult, strengthening their social connections and psychological well-being. Therefore, both family support and community services are crucial factors for the health of the older adult. This study also confirms that older adult individuals with advanced age and disability who receive daily family support and frequent community services experience better improvements in their physical and mental health.

Based on the above results, the study conducted further multivariate analysis, revealing that younger age, absence of chronic diseases, higher economic status, daily family support, frequent community services, and the use of smart healthcare devices can influence the physical health of older adult individuals with advanced age and disability. Additionally, the absence of chronic diseases, urban residence, higher economic status, daily family support, and frequent community services can also affect their psychological well-being. According to the research findings, the main needs of older adult individuals with advanced age and disability in the context of smart healthcare include chronic disease management, accessibility to medical services, emotional support, community services, family support, economic support, and the use of smart healthcare devices. Providing regular health check-ups, chronic disease management services, and personalized health intervention plans to this population can have a certain effect on improving chronic diseases (27). The lack of richness in rural medical resources suggests the need to strengthen the construction of rural medical infrastructure, enhance the accessibility and quality of medical services, and ensure that older adult individuals in rural areas can access timely and high-quality medical services (28). Family companionship and communication, along with diverse community activities and services, are also key factors in improving the psychological well-being of older adult individuals. Given that older adult individuals with advanced age and disability require relatively high expenditures for disease management, economic security is essential (29). Therefore, it is necessary to improve the social security system, increase the coverage and level of old-age pensions, medical insurance, and long-term care insurance, and alleviate the economic burden on the older adult (30). Furthermore, although smart healthcare has been applied to some extent in health management, its popularity is not yet fully comprehensive. Challenges such as the cost of smart healthcare devices and services, lack of internet access and related equipment, insufficient skills and knowledge, and a lack of basic health management knowledge still limit the adoption of smart healthcare in low-income and low-education groups (31). Therefore, it is necessary to address these challenges effectively by implementing measures such as providing economic support, affordable internet services, skills training, health education, and community service points. These initiatives aim to assist low-income and low-educated groups in better utilizing smart healthcare, thereby enhancing their levels of health management and quality of life. This endeavor not only aids in narrowing the health gap but also establishes a foundation for the comprehensive dissemination and adoption of smart healthcare. Additionally, the literature by Li et al. (32) also provides theoretical support for the field of smart healthcare, helping researchers understand the potential and challenges of smart healthcare systems, especially in terms of resource allocation, technological innovation, and data-driven decision-making.

## 6 Conclusion

Based on the above analysis, the older adult population with disabilities who use smart healthcare services are mainly concentrated among those who are married (with a living spouse), residing in urban areas, with an educational level of junior high school or above, and having social security. These groups tend to have better physical health conditions, which are closely related to factors such as younger age, absence of chronic diseases, higher economic status, daily family support, frequent community services, and regular use of smart healthcare devices. Specifically, the older adult with disabilities who are married and have a living spouse generally have better health conditions, indicating that family support plays a positive role in the use of smart healthcare and health management. Additionally, the older adult living in urban areas and with higher educational levels have a higher degree of participation in smart health management, which may be related to better urban infrastructure, higher levels of smart technology penetration, and better awareness of health management. Meanwhile, the study results also show that social support, economic conditions, and the utilization of medical resources have a significant impact on the physical and mental health of the older adult with disabilities. The physical and mental health status of the older adult with disabilities is closely related to their social support network, economic status, and living environment. Therefore, further strengthening social support, improving the economic level of the older adult, and optimizing the allocation of medical resources are important ways to improve the physical and mental health of this group. However, the study still has the following limitations: First, the analysis of differences among different subgroups in the sample (such as gender, region, and educational level) is relatively brief and fails to fully demonstrate the impact of these factors on health status, especially the impact of urban–rural differences and cultural background on the frequency of smart healthcare use. Second, the study mainly adopts a cross-sectional design, which only reveals the factors affecting the health status of the older adult with disabilities in the current state and cannot reflect the dynamic changes and long-term impact of these factors on health status. Third, although the study clearly identifies the health problems of the respondents, it lacks sufficient description of the organizational methods of community nursing services and the actual effectiveness of the combination with smart healthcare technologies. Therefore, future research can further refine the analysis of different subgroup characteristics (such as gender, region, and educational level), adopt a longitudinal design to assess the long-term impact of smart healthcare technologies and social support, and economic conditions on the physical and mental health of the older adult with disabilities. Meanwhile, research also needs to strengthen the exploration of community nursing service models and combine the specific practices of smart healthcare technology applications to provide more in-depth theoretical and practical guidance for future health management.

## Data Availability

The data analyzed in this study is subject to the following licenses/restrictions: the data are from China Family Panel Studies (CFPS), funded by Peking University and the National Natural Science Foundation of China, and maintained by the Institute of Social Science Survey of Peking University. Restrictions apply to the availability of these data, which were used under licence for this study. Requests to access these datasets should be directed to CFPS, Peking University, https://opendata.pku.edu.cn/.
